# The complete mitochondrial genome of *Monochamus alternatus alternatus* (Coleoptera: Cerambycidae)

**DOI:** 10.1080/23802359.2020.1788439

**Published:** 2020-09-18

**Authors:** Qiliao Liao, Xiaozhen Yang, Jiayi Ma, Liangjing Sheng, Shuangquan Zou

**Affiliations:** a College of Forestry, Fujian Agriculture and Forestry University, Fuzhou, China; b Key Laboratory of Integrated Pest Management in Ecological Forests, Fujian Agriculture and Forestry University, Fuzhou, China; c Fujian Colleges and Universities Engineering Research Institute of Conservation and Utilization of Natural Bioresources, College of Forestry, Fujian Agriculture and Forestry University, Fuzhou, China

**Keywords:** Complete mitochondrial genome, *Monochamus alternatus alternatus*, phylogenetic analysis

## Abstract

*Monochamus alternatus alternatus* is the major vector of pinewood nematode, *Bursaphelenchus xylophilus*, in Asia. The length of the complete mitochondria genome of *M. alternatus alternatus* was 15,880 bp with 21% GC content, including 39.7% A, 12.3% C, 8.7% G and 39.3% T. There were 13 protein-coding genes, 22 tRNAs, 2 rRNAs, and one AT-rich region. This study provides a useful genetic information for subsequent study of the differences between *M. alternatus* subspecies.

*Monochamus alternatus* is one of the trunk borer of pine trees and is also the main transmission vector of pinewood nematode that is the agent of the pine wilt disease (Nickle et al. 1981). The study have divided the beetle into two subspecies, including *Monochamus alternatus alternatus* Hope from China and Vietnam, and *Monochamus alternatus endai* Makihara from Japan and South Korea (Makihara 2004). However, because the relevant genomic analysis of each subspecies is not detailed enough at present, the relationship between them in genetic evolution in not clear. We determined the complete mitochondria genome of *Monochamus alternatus alternatus* that from Vietnam in this study. The results could provide an important genetic information to study the genetic evolution of *M. alternatus* subspecies.

The *M. alternatus alternatus* adults were collected from Dingli county, Liangshan province, Vietnam (107°03′50″E, 21°35′54″N) by the traps with sexual attractants. The specimens were stored in the Fujian Agriculture and Forestry University (MA-201912). The total DNA was extracted by Insect DNA Kit (Omega Bio-Tek, GA, USA) and sequenced by the Illumina Hiseq 2500 (Illumina, CA, USA) at the Novogene (Beijing, China). About 48,469,116 reads were assembled by GetOrganelle (Jin et al. 2020). The quality of mitogenome assembly showed that the customized error rate was 0.0059 ± 0.0086. Then, the mitogenome was annotated by GeSeq (Tillich et al. 2017), and manually adjusted in Geneious (Kearse et al. 2012) according to *M. alternatus* (Genbank Accession No. KJ809086.1). The complete mitogenome sequence of *M. alternatus alternatus* has been submitted to NCBI Genbank with accession number MT547196.

The complete mitochondria genome length of *M. alternatus alternatus* was 15,880 bp with 39.7% A, 39.3% T, 12.3% C, and 8.7% G, and the GC content was 21%. The complete mitochondria genome encoded 13 protein-coding genes, 22 tRNAs, 2 rRNAs, and one AT-rich region. The content and order of these genes in the mitochondrial genome of *M. alternatus* were consistent with those of other coleopteran insects (Kim et al. 2009). The complete mitochondrial genome of Japanese pine sawyer, *Monochamus alternatus* have been analyzed, and the content and order of code genes were same between Japanese pine sawyer and *M. alternatus alternatus alternatus* in this study (Li et al. 2016).

According to the mitochondrial genome sequence of *M. alternatus alternatus*, the sequence alignment was performed with the MAFFT (Katoh and Standley 2013). The phylogenetic analysis was constructed with 15 different species of coleoptera by MEGA 6.0 using neighbor-joining tree model with 1000 bootstrap replicates. The result showed that the *M. alternatus alternatus* and Japanese pine sawyer were clustered together, and sister to *Anoplophora glabripennis* (Figure 1). The complete mitochondrial genome of *M. alternatus alternatus* will provide useful genetic information to study the genetic evolution of *M. alternatus* subspecies, as well as in insects of *Monochamus*.

**Figure 1.**
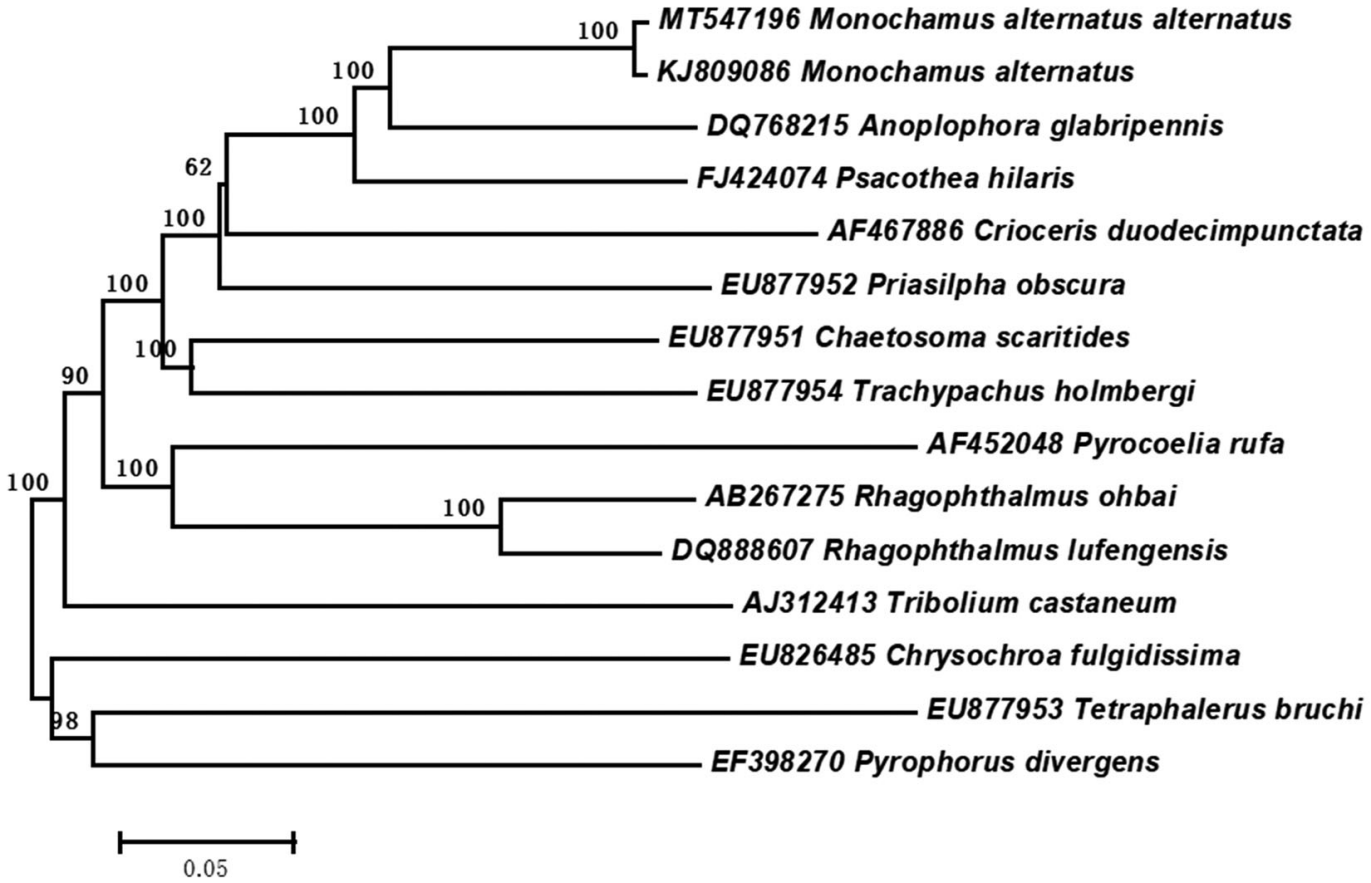
Neighbor-joining tree of the *Monochamus alternatus alternatus* and related 14 different species of coleoptera based on the genome sequence. Numbers labeled on the branch are bootstrap values.

## Data Availability

The data that support the findings of this study are openly available in GenBank of NCBI at https://www.ncbi.nlm.nih.gov, reference number MT547196.
